# Effect of Excessive or Restrictive Energy on Growth Performance, Meat Quality, and Intramuscular Fat Deposition in Finishing Ningxiang Pigs

**DOI:** 10.3390/ani11010027

**Published:** 2020-12-25

**Authors:** Jiayi Chen, Fengming Chen, Xue Lin, Yaodong Wang, Jianhua He, Yurong Zhao

**Affiliations:** College of Animal Science & Technology, Hunan Agricultural University, Changsha 410128, China; jiayi@stu.hunau.edu.cn (J.C.); cfming@stu.hunau.edu.cn (F.C.); risca@tanke.com.cn (X.L.); sxllwyd@gmail.com (Y.W.)

**Keywords:** energy, meat quality, intramuscular fat deposition, Ningxiang pig

## Abstract

**Simple Summary:**

The present study indicated that excessive energy enhanced fat deposition by upregulating expression of lipogenic genes in the longissimus thoracis of a Chinese fat-type pig. In addition, impairment in meat quality resulted in reduced meat tenderness and increased cross-sectional area of muscle fiber aroused by promoting glycolytic muscle fibers differentiation in muscles. These results provided a new perspective on the energy needs of Ningxiang pigs.

**Abstract:**

This study investigated the effects of excessive or restrictive energy on growth performance, meat quality, intramuscular fat (IMF) deposition, and related gene expression in finishing Ningxiang pigs. A total of 36 Ningxiang pigs (43.26 ± 3.21 kg) were randomly assigned to three treatments (6 pens of 2 piglets per treatment) and fed by one of three dietary treatments until the pigs of each treatment weighed approximately 75 kg equally. The three treatments were control diet (digestible energy, DE:13.02 MJ/kg, CON), excessive energy diet (DE 15.22 MJ/kg, EE), and restrictive energy diet (DE 10.84 MJ/kg, RE). Results showed that EE improved average daily gain (ADG) and feed conversion ratio (FCR) (*p* < 0.01), while nothing significantly changed by RE except FCR increasing (*p* < 0.01). EE increased the content of IMF and triglycerides (TG) (*p* < 0.05), L*_24h_ and b*_45min_ (*p* < 0.01), while decreasing cooking loss and meat tenderness in longissimus thoracis (LT) (*p* < 0.05). b*_24h_ was significantly increased with the increase of energy level (*p* < 0.01). Meanwhile, EE increased the cross-sectional area (CSA) of muscle fiber and the mRNA expression of myosin heavy chain (*MyHC*) *IIb*, acetyl CoA carboxylase (*ACC*), fatty acid synthase (*FAS*), and adipocyte fatty-acid binding protein (*FABP4*) (*p* < 0.05). In addition, throughout: A diet supplemented with excessive energy promoted IMF deposition by positively changing lipogenic potential while decreasing tenderness by increasing glycolytic muscle fibers, which together affected meat quality. In terms of growth performance and meat quality, the present study suggests that the low-energy diet is suitable for finishing Ningxiang pigs.

## 1. Introduction

In animals, adipogenesis firstly occurs in visceral fat, followed by subcutaneous and intermuscular, and then is deposited in intramuscular fat last [1]. It is well known that the content of intramuscular fat (IMF) has a positive effect on the sensory quality of pork, while lower fat leads to a decrease in the pork taste [2]. IMF deposition is positively correlated with the percentage of oxidized muscle fibers but negatively correlated with glycolytic muscle fiber in the muscle [3]. It is generally believed that local pig breeds contain more oxidized muscle fibers, thus accounting for better meat quality but slow growth rate, while foreign pig breeds are known for their fast growth rate and high meat production but low meat quality due to containing more glycolytic fibers [4,5,6,7]. In addition to the genetic factor, the nutritional level affects the composition of muscle fiber types and IMF deposition. It has been reported that dramatic changes in fiber type composition (FTC) could be achieved by altering energy and nutrient balance during animal growth [8,9]. It is widely accepted that fat deposition is greater in muscles with increasing energy levels [10].

The Ningxiang pig, as a fat-type local pig species in China, is famous for its delicious taste, which has more generous intramuscular fat (IMF), lower lightness, and higher redness than meat from the improved commercial pig [11]. Distinct from high-performing pigs, Ningxiang pig has a greater propensity to deposit fat due to genetics. Therefore, from the view of feed conversion efficiency, it is not suitable to provide a high-energy diet for finishing Ningxiang pigs. However, in fact, to pursue feed efficiency, the digestible energy level of domestic commercial diet in finishing Ningxiang pigs is so high that it tends to produce fatter carcasses.

Furthermore, dietary energy plays a bidirectional role in regulating the yield and quality of pork. A clear knowledge of the relationships between dietary digestible energy (DE) density and meat quality is necessary in commercial practice. However, previous studies about dietary energy concentration are mainly focused on the growth performance of swine, especially for fat-type pig species, including Ningxiang pig. In addition, the effect mechanism of energy on meat quality and fat metabolism in fat-type pig species receives much less attention at present. Given the foregoing, the Ningxiang pig was used as an animal model to investigate its growth and efficiency, as well as meat quality response to the extreme energy level at the optimal slaughter weight [12,13]. In addition, the present study was conducted to use excessive and restrictive energy to explore whether energy affects fat deposition and meat quality by changing lipid metabolism and/or myofiber characteristics of the muscle. In addition, this study hopes to provide a new experimental basis for the nutritional needs of Ningxiang pigs.

## 2. Materials and Methods

### 2.1. Animals and Diets

This experiment was conducted on a commercial research pig farm (Changsha, China) and was approved by the Animal Care and Use Committee of the Institute of Hunan Agricultural University. A total of 36 (43.26 ± 3.21 kg) Ningxiang pigs (half male, half female) were randomly assigned to 1 of 3 treatments (6 pens of 2 piglets per treatment). The 3 diets were control diet (CON, DE: 13.02 MJ/kg), excessive energy diet (EE, DE: 15.22 MJ/kg), and restrictive energy diet (RE, DE: 10.84 MJ/kg). All diets were formulated to meet the recommendations of the Chinese National Feeding Standard Type 2 for fatty growing pigs (2004) and contained similar contents of crude protein (CP) and amino acids, as shown in Table 1. Pigs were fed 3 times per day by a rationed feeding with free access to water. The feeding experiment continued until pigs of each treatment weighed approximately 75 kg equally. The feed intake was recorded weekly and calculated for each pen. Pigs were weighed at the beginning and the end of the experiment, individually.

### 2.2. Slaughter Procedure and Sample Collection

One pig with close to average weight was selected from each pen and fasted for 12 h. Pigs were slaughtered by standard commercial procedures. Samples of longissimus thoracis (LT) were immediately collected from the right side of the carcass, some stored at 4 °C for meat quality evaluation and others frozen at −20 °C for muscle chemical analysis. LT samples for RNA extraction were taken and treated by liquid nitrogen, and then stored at −80 °C for further analysis. Fresh samples of LT (1 cm^3^) were excised and put in 4% paraformaldehyde in PBS (pH 7.3) for further morphological detection. 

### 2.3. Meat Quality Index Measurements

Meat color (lightness L*, redness a*, and yellowness b*) were determined in triplicate at 45 min and 24 h by reflectance spectrophotometer (CR-410, Kinica Minolta Sensing Inc., Osaka, Japan). Water-holding capacity was tested using drip loss. Briefly, a 2 cm-thick LT sample was weighed. After being hung in a sealed plastic container for 24 h at 4 °C, the samples were taken out and weighed again. Drip loss was expressed as the difference (%) from the initial sample weight, based on Honikel (1998) [14]. The weight-recorded muscle sample was placed in a pot and steamed until the internal temperature reached 70 °C. After being cooled for 30 min and dried with a paper towel, a percentage compared to the initial weight was used to calculate the cooking loss. For tenderness, a minimum of 5 meat pieces parallel to the muscle fiber direction was sheared in the Warner–Bratzler device. The shear value was recorded (expressed in N), and the averages were calculated to determine the shear force value per sample. 

### 2.4. Meat Chemical Composition and Biochemical Parameters Analysis 

Total moisture, CP and IMF of LT Samples were analyzed referring to the Association of Analytical Chemists methods (AOAC 2000) [15]. Triglyceride (TG) was measured using a TG kit (Nanjing Jiancheng Bioengineering Institute, Nanjing, China) according to the manufacturer’s protocol. 

### 2.5. Muscle Histological Analysis 

LT muscle samples were cut perpendicularly to the direction of the myofibers in cryostat (Leica CM1850) and then stained with classic hematoxylin and eosin staining. The sections were magnified 400x under light microscopy (Olympus, Tokyo, Japan), and 8 views were captured in each section. The total fiber number (TFN) was measured by the Image-Pro Plus 4.5 software (Media Cybernetics Inc., Shanghai, China). The cross-sectional area (CSA) of muscle fiber in LT was estimated by extrapolation from the number of fibers counted over the selected fields, and the total fiber number (TFN).

### 2.6. Quantitative Real-Time PCR Analysis 

Total RNA was extracted from samples with TRIZOL reagent (Beijing Solarbio Science & Technology Co., Ltd., Beijing, China); the purity and integrity of RNA were detected by Nanodrop (Thermo Scientific, Waltham, America) and electrophoresis in 1% agarose gel, respectively. Approximately 1.0μg of total RNA was reverse-transcribed with RT Reagents (Hunan Accurate Biotechnology Co., Ltd., Changsha, China). The primers were designed via Primer 5.0 software, as shown in Table 2. *β-actin and GAPDH* were chosen as the endogenous control gene. The PCR cycling condition was as followed: 95 °C for 10 min, 40 cycles at 95 °C for 15 s and 60 °C for 60 s, and 1 cycle at 72 °C for 30 s. The relative expression was expressed as a ratio of the target gene to the control gene according to the 2 ^−(∆∆Ct)^ method, as described previously [16]. 

### 2.7. Muscle Hydrolyzed Amino Acids Analysis 

0.1 g freeze-dried and crushed muscle samples, mixed with 10 mL 6 M hydrochloric acid, were hydrolyzed at 110 °C for 22 h. The hydrolyzed mix was filtered to a constant volume, evaporated, diluted, and filtered with a 0.22 μm filter membrane, and then analyzed by the high-speed amino acid analyzer (L-8900, Shimazi, Japan). 

### 2.8. Statistical Analysis 

All data were performed using the one-way analysis of variance (ANOVA) to test homogeneity of variances via Levene’s test and followed with Duncan’s method (IBM SPSS 23 software, Chicago, IL, USA). Results were expressed as the mean ± SEM. The level of statistical significance was set at *p* < 0.05.

## 3. Results

### 3.1. Growth Performance

The pigs in different groups were slaughtered at a similar finishing weight according to the experimental plan (*p* > 0.05; Table 3). No effect of dietary treatment was detected on carcass weight or slaughter yield (*p* > 0.05). The average daily gain (ADG) of the EE group was higher than that of the other two groups (*p* < 0.01). In addition, the feed conversion ratio (FCR) was significantly decreased with the increase of energy level (*p* < 0.01).

### 3.2. Muscle Chemical Composition and Biochemical Parameters

Excessive or restrictive energy showed no difference in the content of total moisture and CP in LT (*p* > 0.05). However, excessive energy significantly increased the contents of IMF and TG in LT (*p* < 0.05; Table 4).

### 3.3. Meat Quality

Table 5 presents that excessive energy had an apparent impact on cooking loss, shear force, and meat color. Cooking loss of the EE group was significantly reduced (*p* < 0.05), while the shear force of the EE group was significantly increased compared with the other two groups (*p* < 0.01). L*_24h_ of the EE group was higher than that of the other two groups (*p* < 0.01). b*_45min_ of the RE group was markedly lower than that of the other two groups (*p* < 0.01). b*_24h_ was significantly increased with the increase of energy level (*p* < 0.01).

### 3.4. Muscle Fiber Morphology

**Figure 1:**
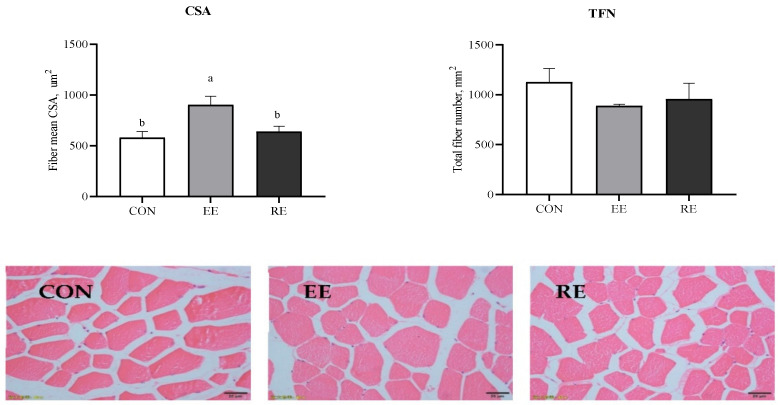
Morphological section of LT muscle (magnification, ×400). CON, control diet group. EE, excessive energy diet group. RE, restrictive energy diet group. Values with different letters are significantly different among the three groups (*p* < 0.05). Upper: Morphology analysis of total fiber number (TFN) and fiber cross-sectional area (CSA) in longissimus thoracis (LT) of finishing Ningxiang pigs fed with excessive energy or restrictive energy.

Excessive energy markedly enhanced fiber mean CSA in LT (*p* < 0.05). However, there were no significant differences in TFN among the three groups (*p* > 0.05; Figure 1).

### 3.5. MyHC Expression and IMF Deposition Related Gene Expression Levels

**Figure 2:**
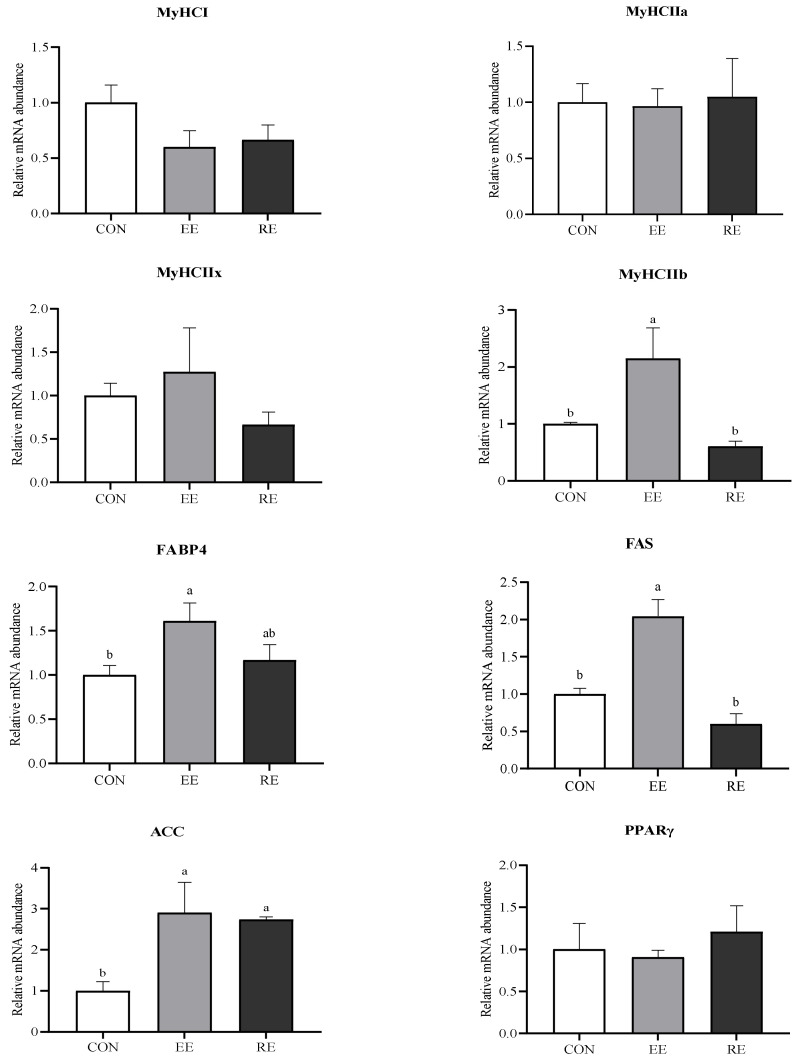
Genes expression of longissimus thoracis (LT) in finishing Ningxiang pigs fed with excessive energy or restrictive energy dietary. Myosin heavy chain (*MyHC*), adipocyte fatty-acid binding protein (*FABP4*), fatty acid synthase (*FAS*), acetyl CoA carboxylase (*ACC*), and peroxisome proliferator-activated receptor γ (*PPARγ*). CON, control diet group. EE, excessive energy diet group. RE, restrictive energy diet group. Values with different letters are significantly different among the three groups (*p* < 0.05).

The mRNA expression of *MyHC I, IIa*, and *IIx* in LT were not affected by dietary energy level, but the mRNA expression level of *MyHC IIb* was upregulated in the EE group (*p* < 0.05; Figure 2), as well as the mRNA expression of *FABP4* and *FAS* (*p* < 0.05). acetyl CoA carboxylase (*ACC*) mRNA expression was upregulated in both the EE and RE group (*p* < 0.05) while there was no difference in the mRNA expression of *PPARγ* among the three groups (*p* > 0.05).

### 3.6. Muscle Hydrolyzed Amino Acid

Excessive energy reduced the proline (Pro) content (*p* < 0.01), but increased Cysteine (Cys) content (*p* < 0.01; Table 6). No apparent differences were observed in other amino acids among the three groups.

## 4. Discussion

The dietary energy concentration is a primary determinant to growth performance and costs most in the diet. It is generally accepted that increasing dietary energy concentration can improve growth performances [17] and provide fatter carcasses [18]. To our knowledge, information available in the previous literature on the response of the pigs to changes in dietary energy density is inconclusive, which may depend on the developmental stage of the pigs. In the grower stage, pig growth increments alongside dietary energy, indicating that pigs were in an energy-dependent phase of growth [19]. However, in finishing pigs, reducing the energy concentration might reduce ADG or increase FCR without affecting carcass quality [20,21]. The current trial also was observed similar results that excessive energy improved ADG and FCR, while nothing changed with restrictive energy except increased FCR. There are reports showing that it is comparable in feed efficiency and growth performance of lean-type pigs fed different energy densities [22,23]. Similarly, in the present study, RE was comparable to CON in terms of effects on ADG and slaughter yield, suggesting that a restrictive energy diet may have some beneficial effects on carcass weight in compensation to its reduced fattening and feed efficiency. Properly decreasing dietary energy in Ningxiang pigs is feasible to save feed resources, particularly if it tolerates rough feeding.

Meat quality can be assessed scientifically by some traits, including composition, nutrients, colorants, water-holding capacity (WHC), tenderness, flavor, and IMF. In general, color indicates its primary acceptability to consumers, while tenderness is rated as the most critical palatability trait for cooked meat, followed by flavor and juiciness [24]. High IMF content is associated with improved eating quality of meat [25]. The percentage of IMF required in meat raised for a pleasant eating experience is at least 1.5% commonly, and 2% to 3% for optimal eating quality [26]. The major finding of the present study was that the IMF content of the EE group was far more than 3% and much higher than that of the other two groups. Different from the results of Heyer et al. (2007) [27] and Skiba et al. (2010) [28], we did not observe the effects of restrictive energy on IMF contents, which may be due to the unique breed of Ningxiang pig that itself has high IMF content. However, the contents of IMF and TG in LT muscle increased in the excessive energy group. Indeed, dietary energy content regulates lipid accumulation, especially IMF [10,29]. Furthermore, excessive energy has a greater impact on meat quality and IMF than restrictive energy in this study. One possible reason may be that an excessive energy diet increased the amount of energy available for fat deposition, resulting in higher IMF content [30].

In the present study, both reduced cooking loss and enhanced IMF content of the EE group were observed, which was consistent with the result of Lebret et al. (2008) [31] and Hocquette et al. (2010) [2]. Thus, it can be seen that meat with high IMF content has improved juiciness [32] after relatively long-heating in a moist environment [8]. Besides, meat from the EE group had higher b* values, possibly due to its higher IMF content [30]. Chartrin et al. (2006) [33] also found that duck meat with high IMF content due to overfeeding indicates greater yellowness. Nevertheless, muscles with a higher L* value exhibit a higher percentage of type IIB number [3,34,35], which may be reflected in the present study as well. Regarding meat tenderness, it was evaluated by shear force. It is well known that shear force increases along with dietary DE concentration decreasing [36,37]. Alternatively, energy or protein restriction negatively influenced meat tenderness [28,38,39]. However, there are reports showing that meat tenderness was not affected by a restrictive diet [40], which likewise occurred in current study. Unexpectedly, excessive energy decreased meat tenderness, which is another major finding from the present study. In general, higher meat tenderness is associated with higher IMF content [3,41]. Interestingly, excessive energy increased IMF content but had no positive impact on meat tenderness in our study, which is similar to the results of Van Laack et al. (2001) [42]. Teye et al. (2006) [43] also reported that there were no differences in meat tenderness between fed restricted and fed non-restricted animals with different IMF content. These results indicated the difference in IMF content among groups was not positively reflected at shear force as usual.

As previously stated, increased IMF content by excessive energy failed to contribute to meat tenderness, and there may be other factors also influencing shear force, such as muscle fiber condition. Morphology traits such as TFN, CSA, and FTC are major determinants of meat quality, especially tenderness [44,45]. It is commonly stated that meat tenderness will be impaired by increased CSA [46]. Here EE pigs presented increased CSA compared with CON pigs, which was consistent with their reduced tenderness. It is well documented that muscles with a larger fiber size, primarily type IIB fiber, exhibit tougher meat than that of smaller fiber size in cattle [47] and pig [8,48]. The possibility is that the size of muscle fibers affects muscle growth potential and the size of the fiber bundle, resulting in the visible coarseness of transverse sections of meats [8].

CSA is mainly related to the type IIb fibers as a consequence of the abundance of type IIb fibers in LT [49]. There are four types of fiber in skeletal muscle, type I, IIa, IIx, and IIb, whose metabolic type transitions from oxidation to glycolysis and which transformed according to the law of I ↔ IIa ↔ IIx ↔ IIb. In pigs, a correlation between the glycolytic muscle fiber percentage and the pale, soft, exudative meat condition has been known [50,51], and a negative impact of glycolytic muscle fibers and high glycolytic metabolism on meat tenderness has been reported [52]. On the other hand, glycolytic fibers exhibit a larger CSA than oxidized fibers [53,54,55]. Since it has been observed that excessive dietary energy increased CSA with regards to decreased tenderness and the known higher glycolytic muscle fibers are associated with larger CSA and lower tenderness. Therefore, we hypothesis that excessive energy reduces tenderness by increasing glycolytic muscle fibers differentiation in muscles. Accordingly, the fiber type composition was analyzed. As we expected, *MyHC IIb* mRNA expression in LT was upregulated in the EE group, indicating that excessive energy could induce a muscle fiber transition toward more glycolytic muscle fibers. Consistently, Solomon and Lynch (1988) [56] found that the longissimus muscles from lambs fed the lower-energy diet contained more type I and fewer type IIB fibers. As well, our study showed fewer type IIB fibers in the restrictive energy group but not significantly. The possibility could be involved in the muscle-specific expression of the MyHC gene regulated by energy level. According to Harrison et al. (1996) [57], when the body is in a state of undernutrition, it will selectively increase oxidized muscle fibers during the period of myofiber hypertrophy, as compared with glycolytic muscle fibers, oxidized muscle fibers require less energy to produce the same tension. Additionally, it is also due to its own smaller CSA that oxidized muscle fibers require less energy. In turn, this result easily occurred in the current study, where glycolytic muscle fibers were selectively increased when supplied with excessive energy. Altogether, the transition of fiber types, along with the increased CSA, may mainly explain that excessive energy reduces meat tenderness.

IMF is the amount of fat deposited inside the muscle, influencing the energy metabolism of skeletal muscle, as well as meat quality [58]. TG, the major component of IMF in muscles, derived from the synthesis or the de novo synthesis of fatty acids in the circulatory system, determines the extent of IMF deposition [59]. As the content of IMF and TG in LT markedly increased in the EE group, the relative mRNA expression levels of *ACC* and *FAS* were assessed and found to be upregulated. Under the action of ACC, Acetyl-CoA is carboxylated to malonyl-CoA, a two-carbon unit for the synthesis of long-chain fatty acids [60]. FAS catalyzes small molecular carbon units to long-chain fatty acids [61], which was observed to have the strongest correlation to IMF content among the chosen 30 lipid metabolism genes by Wang et al. (2020) [62]. In the present study, the upregulated expression of these lipogenic genes in the EE group indicated that there was an increased capacity for de novo synthesis of FFA due to energy excess diet, leading to an increase in IMF accumulation [10]. Considering the high inclusion of soy oil in the EE diet, it has been shown previously that fatty acids regulate lipid metabolism via altering gene expression of lipogenic enzymes in the liver and adipose [63]. However, meat quality characteristics are unlikely to be affected via an altered dietary composition of phospholipids [64]. The extent of fatty acids in tissue concentration altered from dietary fat type depends on the dietary energy level [65]. According to Fang et al. [66], the inclusion of 0%, 3.17%, and 10.50% soy oil in the diet, the DE level was 12.82, 14.24, and 15.66 MJ/kg, respectively, which had no impact on meat quality. Therefore, it is reasonable to suggest that the different energy levels played a major impact in the present study.

FABP4, considered candidate genes for IMF deposition [67,68], is largely involved in regulating fatty acid intake and intracellular transporting [69]. The upregulated mRNA expression of *FABP4* in the EE group may explain higher IMF content due to the efficient transport of fatty acids towards lipid synthesis. Alternatively, the higher expression of *FABP4* mRNA will deliver more fatty acid to mitochondrial oxidation [70,71]. Coincidentally, the present results, which showed a higher proportion of MyHC IIb, *FABP4* abundance, and IMF content in the EE group, were consistent with the fact that glycolytic muscle fibers were less oxidative and used fewer lipids as fuel substrate than other fiber types. Collectively, these observations of the present study suggested that lipid anabolic pathways, intracellular trafficking of fatty acids supported by FABP, and myofiber energetic metabolism may contribute an explanation of IMF content increasing caused by excessive energy.

Finally, hydrolyzed amino acids in LT were determined to evaluate the nutritional value of meat from Ningxiang pigs. A food protein with high nutritional value should contain comprehensive and well-proportioned essential amino acids (EAA), and it is best to be close to or consistent with the nutritional needs of the human body thus that EAAs will be absorbed thoroughly. These findings in all groups in this study fit the FAO/WHO/UNU (Joint, 1985) [72] essential amino acid benchmark model, where EAA/TAA should be around 40%, and EAA/NEAA should be over 60% in the amino acid composition of high-quality protein. Cys and Pro, called functional amino acids, were increased and decreased, respectively, in the excessive energy group. Cys is considered capital contributors to meat flavor development [73]. Pro is a sweet amino acid, and its metabolite (hydroxyproline) is closely related to meat tenderness [74]. Taken together, Ningxiang pigs retained a variety of amino acids regardless of fed excessive energy or restricted energy, which offered high nutritional value. As a local, high-quality pig breed resource, it has development potential. Moreover, judging from increased Cys and decreased Pro, excessive energy was conducive to meat flavor formation and meat tenderization to a certain extent in the present study.

## 5. Conclusions

RE is comparable to CON in terms of growth performance and meat quality of Ningxiang pigs, suggesting the acceptability to lower dietary energy in Ningxiang pigs. Excessive energy led to the growth promotion of finishing Ningxiang pigs but tended to affect its meat quality negatively. Excessive energy in this study promoted IMF deposition by changing lipogenic potential to affect meat quality while causing a decrease in tenderness by increasing CSA and glycolytic muscle fibers, in particular, MyHC IIb. In addition, excessive energy had a greater impact on meat quality and IMF than restrictive energy, or rather, a more negative impact. The trial of extreme energy levels on the IMF deposition and myofiber characteristics may clarify the functional role of energy in the regulation of meat quality and provide a new perspective for the energy needs of Ningxiang pigs.

## Figures and Tables

**Table 1:** Ingredient and chemical composition of experimental diets. (air-dry matter basis) ^1^.

Ingredient, %	CON	EE	RE
Corn	54.90	57.10	47.90
Soybean meal	8.20	5.60	8.20
Peanut meal	0.00	5.00	0.00
Wheat bran	21.30	0.00	29.8
Rice bran	6.40	21.40	0.00
Soybean oil	3.20	7.90	0.00
CaHPO_4_	0.27	0.51	0.19
Limestone	1.01	0.90	1.05
L-Lys HCl 98%	0.11	0.11	0.13
Threonine	0.01	0.00	0.03
Tryptophan	0.00	0.02	0.00
Rice chaff	1.7	0.46	1.5
Zeolite	1.90	0.00	10.20
Premix ^2^	1.00	1.00	1.00
Total	100.00	100.00	100.00
Nutrient levels ^3^			
Digestible energy, MJ/kg	13.02	15.22	10.84
Crude protein, %	12.01	12.01	12.01
Crude fiber, %	4.07	3.31	4.06
Crude fat, %	5.28	9.40	2.12
Lys, %	0.60	0.60	0.60
Met, %	0.20	0.20	0.20
Thr, %	0.44	0.44	0.44
Try, %	0.13	0.12	0.13
Calcium, %	0.50	0.50	0.50
Total phosphorus, %	0.53	0.61	0.49
Available phosphorus, %	0.16	0.16	0.16

^1^ Basal diet formulated according to the Chinese National Feeding Standard for Swine; ^2^ supplied, per kilogram of diet: 0.05 mg Cu; 0.03 mg I; 0.8 mg Fe; 0.002 mg Se; 0.8 mg Zn; 0.06 mg Mn; 1.3 mg vitamin K (menadione); 2 mg vitamin B1; 5.8 mg vitamin B2; 18.79 mg vitamin B3; 14.5 μg vitamin B12; 3324 IU vitamin A; 376 IU vitamin D; 28.9 IU vitamin E; 80 mg choline chloride; 200 mg antioxidants; 500 mg Fungicide; ^3^ The contents of digestible energy, crude protein, crude fiber, calcium, and total phosphorus were analyzed.

**Table 2:** Primers used for quantitative real-time PCR.

Gene	Accession No.	Primer Sequence (5′-3′)	Product Size (bp)
*MyHC I*	AB053226	F: AAGGGCTTGAACGAGGAGTAGA	115
		R: TTATTCTGCTTCCTCCAAAGGG	
*MyHC IIa*	AB025260	F: GCTGAGCGAGCTGAAATCC	137
		R: ACTGAGACACCAGAGCTTCT	
*MyHC IIx*	AB025262	F: AGAAGATCAACTGAGTGAACT	149
		R: AGAGCTGAGAAACTAACGTG	
*MyHC IIb*	AB025261	F: ATGAAGAGGAACCACATTA	166
		R: TTATTGCCTCAGTAGCTTG	
*ACC*	NM-001114269	F: GGCCATCAAGGACTTCAACC	120
		R: ACGATGTAAGCGCCGAACTT	
*FAS*	NM-001099930	F: ACACCTTCGTGCTGGCCTAC	112
		R: ATGTCGGTGAACTGCTGCAC	
*PPAR γ*	NM-214379	F: GAGGGCGATCTTGACAGGAA	124
		R: GCCACCTCTTTGCTCTGCTC	
*β-actin*	XM-003124280.3	F: CCTGCGGCATCCACGAAAC	123
		R: TGTCGGCGATGCCTGGGTA	
*GAPDH*	NM-001206359.1	F: TCGGAGTGAACGGATTTGGC	95
		R: GAAGGGGTCATTGATGGCGA	

**Table 3:** Effects of excessive energy or restrictive energy on growth performance of finishing Ningxiang pigs.

Items	CON	EE	RE	SEM	*p* Value
Initial body weight (kg)	43.75	43.88	42.25	1.10	0.822
Final body weight (kg)	73.92	75.25	69.00	1.48	0.176
Average daily gain (g)	372.43 ^b^	522.92 ^a^	313.27 ^b^	32.21	<0.01
Average daily feed intake (ADFI, kg)	1.74	1.76	1.73	0.05	0.969
Feed conversion ratio (FCR)	4.84 ^b^	3.38 ^c^	5.55 ^a^	0.31	<0.01
Duration of fattening (days)	81	60	81	/	/
Carcass weight (kg)	54.72	56.98	51.22	1.02	0.089
Slaughter yield (%)	73.95	73.43	72.34	0.71	0.678

Values in the same line with different superscripts are significant (*p* < 0.05). CON, control diet group. EE, excessive energy diet group. RE, restrictive energy diet group. IMF, intramuscular fat.

**Table 4:** Effects of excessive energy or restrictive energy on chemical composition and biochemical parameters in longissimus thoracis muscle of finishing Ningxiang pigs.

Items	CON	EE	RE	SEM	*p* Value
Total moisture (%)	74.8	73.56	75.92	0.51	0.226
IMF (%)	2.13 ^b^	4.21 ^a^	1.55 ^b^	0.41	<0.05
CP (%)	22.43	21.25	22.21	0.32	0.326
TG (mmol/L)	0.73 ^b^	2.13 ^a^	1.11 ^b^	0.24	<0.05

Values in the same line with different superscripts are significant (*p* < 0.05). CON, control diet group. EE, excessive energy diet group. RE, restrictive energy diet group. IMF, intramuscular fat. CP, crude protein. TG, triglycerides.

**Table 5:** Effects of excessive energy or restrictive energy on meat quality in longissimus thoracis muscle of finishing Ningxiang pigs.

Items		CON	EE	RE	SEM	*p* Value
Cooking loss (%)		34.45 ^a^	22.05 ^b^	35.06 ^a^	2.17	<0.05
Drip loss (%)		1.61	1.4	1.29	0.18	0.777
Shear force (N/kg)		36.80 ^b^	60.52 ^a^	44.10 ^b^	2.41	<0.05
Lightness	L*_45min_	42.36	42.01	43.74	0.85	0.734
	L*_24h_	43.84 ^b^	54.59 ^a^	45.85 ^b^	1.51	<0.01
Redness	a*_45min_	10.41	10.45	8.18	0.57	0.213
	a*_24h_	11.33	12.81	8.66	0.76	0.105
Yellowness	b*_45min_	5.44 ^a^	5.96 ^a^	4.37 ^b^	0.23	<0.01
	b*_24h_	6.30 ^b^	8.05 ^a^	4.63 ^c^	0.43	<0.01

Values in the same line with different superscripts are significant (*p* < 0.05). CON, control diet group. EE, excessive energy diet group. RE, restrictive energy diet group.

**Table 6:** Effects of excessive energy or restrictive energy on Hydrolyzed amino acid in longissimus thoracis muscle of finishing Ningxiang pigs, g/100 g.

Items	CON	EE	RE	SEM	*p* Value
Asp	2.01	1.91	1.97	0.02	0.261
Tyr	0.79	0.79	0.77	0.02	0.925
His	1.04	0.96	1.02	0.02	0.14
Arg	1.36	1.27	1.31	0.02	0.083
Ser	0.88	0.83	0.85	0.01	0.174
Glu	3.6	3.46	3.51	0.04	0.335
Pro	0.71 ^a^	0.63 ^b^	0.68 ^a^	0.01	<0.01
Gly	0.91	0.9	0.88	0.01	0.505
Ala	1.22	1.24	1.19	0.02	0.47
Cys	0.20 ^b^	0.35 ^a^	0.23 ^b^	0.02	<0.01
Val	1.04	1.11	1.02	0.02	0.221
Met	0.66	0.75	0.63	0.03	0.364
Ile	0.98	1.05	0.96	0.02	0.292
Leu	1.75	1.74	1.71	0.03	0.844
Thr	1.00	0.95	0.98	0.01	0.249
Phe	0.89	0.87	0.86	0.02	0.682
Lys	1.97	1.83	1.89	0.02	0.071
TAA	20.98	20.62	20.41	0.26	0.691
NEAA	12.72	12.33	12.38	0.147	0.505
EAA	8.25	8.29	8.03	0.126	0.713
EAA/TAA	39.34	40.19	39.35	0.219	0.219
EAA/NEAA	64.85	67.25	64.89	0.618	0.218

Values in the same column with different superscripts are significant (*p* < 0.05). CON, control diet group. EE, excessive energy diet group. RE, restrictive energy diet group. EAA, essential amino acid. NEAA, non-essential amino acid.

## Data Availability

Data is contained within the article. The data used to support the findings of this study are available from the corresponding author upon request.

## References

[1] Hausman G.J., Dodson M.V., Ajuwon K., Azain M., Barnes K.M., Guan L.L., Jiang Z., Poulos S.P., Sainz R.D., Smith S. (2009). Board-invited review: The biology and regulation of preadipocytes and adipocytes in meat animals. J. Anim. Sci..

[2] Hocquette J.F., Gondret F., Baéza E., Medale F., Jurie C., Pethick D.W. (2010). Intramuscular fat content in meat-producing animals: Development, genetic and nutritional control, and identification of putative markers. Animal.

[3] Hwang Y.H., Kim G.D., Jeong J.Y., Hur S., Joo S. (2010). The relationship between muscle fiber characteristics and meat quality traits of highly marbled Hanwoo (Korean native cattle) steers. Meat Sci..

[4] Yang F.Y., Huang J.X., Zhou X.R., Yao Y., Jiang S., Huan Y., Yang J., Liu Z. (2013). MyHC fiber type composition in Rongchang and Landrace pigs of similar body weight. J. Food Agric. Environ..

[5] Wojtysiak D., Połtowicz K. (2014). Carcass quality, physico-chemical parameters, muscle fiber traits and myosin heavy chain composition of m. longissimus lumborum from Puławska and Polish Large White pigs. Meat Sci..

[6] Zhang C., Luo J.Q., Zheng P., Yu B., Huang Z.Q., Mao X.B., He J., Yu J., Chen J.L., Chen D.W. (2015). Differential expression of lipid metabolism-related genes and myosin heavy chain isoform genes in pig muscle tissue leading to different meat quality. Animal.

[7] Poklukar K., Čandek-Potokar M., Batorek Lukač N., Tomavzin U., Vskrlep M. (2020). Lipid Deposition and Metabolism in Local and Modern Pig Breeds: A Review. Animals.

[8] Joo S.T., Kim G.D., Hwang Y.H., Ryu Y.C. (2013). Control of fresh meat quality through manipulation of muscle fiber characteristics. Meat Sci..

[9] Lee S.H., Joo S.T., Ryu Y.C. (2010). Skeletal muscle fiber type and myofibrillar proteins in relation to meat quality. Meat Sci..

[10] Liu Z.H., Yang F.Y., Kong L.J., Lai C.H., Piao X.S., Gu Y.H., Ou X.Q. (2007). Effects of dietary energy density on growth, carcass quality and mRNA expression of fatty acid synthase and hormone-sensitive lipase in finishing pigs. Asian Austr. J. Anim. Sci..

[11] Feng Z.M., Guo J.P., Kong X.F., Wang W.C., Li F.N., Nyachoti M., Yin Y.L. (2012). Molecular cloning and expression profiling of G protein coupled receptor 120 in Landrace pig and different Chinese indigenous pig breeds. J. Food Agric. Environ..

[12] Jiang Q., Li C., Yu Y., Xing Y., Xiao D., Zhang B. (2018). Comparison of fatty acid profile of three adipose tissues in Ningxiang pigs. Anim. Nutr..

[13] Xing Y., Wu X., Xie C., Xiao D., Zhang B. (2020). Meat Quality and Fatty Acid Profiles of Chinese Ningxiang Pigs Following Supplementation with N-Carbamylglutamate. Animals.

[14] Honikel K.O. (1998). Reference methods for the assessment of physical characteristics of meat. Meat Sci..

[15] Horwitz W. (2000). Official Methods of Analysis of AOAC International.

[16] Yin J., Li Y., Zhu X., Han H., Ren W., Chen S., Bin P., Liu G., Huang X., Fang R. (2017). Effects of long-term protein restriction on meat quality, muscle amino acids, and amino acid transporters in pigs. J. Agric. Food Chem..

[17] Weber T.E., Richert B.T., Belury M.A., Gu Y., Enright K., Schinckel A.P. (2006). Evaluation of the effects of dietary fat, conjugated linoleic acid, and ractopamine on growth performance, pork quality, and fatty acid profiles in genetically lean gilts. J. Anim. Sci..

[18] Hinson R.B., Wiegand B.R., Ritter M.J., Allee G.L., Carr S.N. (2011). Impact of dietary energy level and ractopamine on growth performance, carcass characteristics, and meat quality of finishing pigs. J. Anim. Sci..

[19] De la Llata M., Dritz S.S., Tokach M.D., Goodband R.D., Nelssen J.L., Loughin T.M. (2001). Effects of dietary fat on growth performance and carcass characteristics of growing-finishing pigs reared in a commercial environment. J. Anim. Sci..

[20] Kerr B.J., Southern L.L., Bidner T.D., Friesen K.G., Easter R.A. (2003). Influence of dietary protein level, amino acid supplementation, and dietary energy levels on growing-finishing pig performance and carcass composition. J. Anim. Sci..

[21] Serrano M.P., García L.C., Valencia D.G., Lazaro R., Gorriz M.A.L. (2013). Effect of energy concentration on growth performance and carcass quality of Iberian pigs reared under intensive conditions. Span. J. Agric. Res..

[22] Lee C.Y., Kim M.H., Ha D.M., Park J.W., Oh G.Y., Lee J.R., Ha Y.J., Park B.C. (2007). Effects of the energy level of the finisher diet on growth efficiency and carcass traits of high-market weight pigs. J. Anim. Sci. Technol..

[23] Ha D.M., Kim G.D., Han J.C., Jeong J., Park M., Park B., Joo S., Lee C. (2010). Effects of dietary energy level on growth efficiency and carcass quality traits of finishing pigs. J. Anim. Sci. Technol..

[24] Glitsch K. (2000). Consumer perceptions of fresh meat quality: Cross-national comparison. Br. Food J..

[25] Wood J.D., Nute G.R., Richardson R.I., Whittington F.M., Southwood O., Plastow G., Mansbridge R., da Costa N., Chang K.C. (2004). Effects of breed, diet and muscle on fat deposition and eating quality in pigs. Meat Sci..

[26] Fortin A., Robertson W.M., Tong A.K.W. (2005). The eating quality of Canadian pork and its relationship with intramuscular fat. Meat Sci..

[27] Heyer A., Lebret B. (2007). Compensatory growth response in pigs: Effects on growth performance, composition of weight gain at carcass and muscle levels, and meat quality. J. Anim. Sci..

[28] Skiba G. (2010). Effects of energy or protein restriction followed by realimentation on the composition of gain and meat quality characteristics of Musculus longissimus dorsi in pigs. Arch. Anim. Nutr..

[29] Suarez-Belloch J., Sanz M.A., Joy M., Latorre M.A. (2013). Impact of increasing dietary energy level during the finishing period on growth performance, pork quality and fatty acid profile in heavy pigs. Meat Sci..

[30] Alonso V., del Mar Campo M., Provincial L., Roncales P., Beltran J.A. (2010). Effect of protein level in commercial diets on pork meat quality. Meat Sci..

[31] Lebret B. (2008). Effects of feeding and rearing systems on growth, carcass composition and meat quality in pigs. Anim. Int. J. Anim. Biosci..

[32] Luchak G.L., Miller R.K., Belk K.E., Hale D.S., Michaelsen S.A., Johnson D.D., West R.L., Leak F.W., Cross H.R., Savell J.W. (1998). Determination of sensory, chemical and cooking characteristics of retail beef cuts differing in intramuscular and external fat. Meat Sci..

[33] Chartrin P., Méteau K., Juin H., Bernadet M., Guy G., Larzul C., Remignon H., Mourot J., Duclos M.J., Baeza E. (2006). Effects of intramuscular fat levels on sensory characteristics of duck breast meat. Poultry Sci..

[34] Ryu Y.C., Kim B.C. (2006). Comparison of histochemical characteristics in various pork groups categorized by postmortem metabolic rate and pork quality. J. Anim. Sci..

[35] Kim G.D., Jeong J.Y., Hur S.J., Yang H., Jeon J., Joo S. (2010). The relationship between meat color (CIE L* and a*), myoglobin content, and their influence on muscle fiber characteristics and pork quality. Korean J. Food Sci. Anim. Resour..

[36] Matthews J.O., Higbie A.D., Southern L.L., Coombs D.F., Bidner T.D., Odgaard R.L. (2003). Effect of chromium propionate and metabolizable energy on growth, carcass traits, and pork quality of growing-finishing pigs. J. Anim. Sci..

[37] Zeng Z., Yu B., Mao X., Chen D. (2012). Effects of dietary digestible energy concentration on growth, meat quality, and PPARγ gene expression in muscle and adipose tissues of Rongchang piglets. Meat Sci..

[38] Kristensen L., Therkildsen M., Riis B., Sorensen M.T., Oksbjerg N., Purslow P.P., Ertbjerg P. (2002). Dietary-induced changes of muscle growth rate in pigs: Effects on in vivo and postmortem muscle proteolysis and meat quality. J. Anim. Sci..

[39] Stolzenbach S., Therkildsen M., Oksbjerg N., Lazarotti R., Ertbjerg P., Lametsch R., Byrne D.V. (2009). Compensatory growth response as a strategy to enhance tenderness in entire male and female pork M. longissimus thoracis. Meat Sci..

[40] Batorek N., Škrlep M., Prunier A., Louveau I., Noblet J., Bonneau M., Vcandek-Potokar M. (2012). Effect of feed restriction on hormones, performance, carcass traits, and meat quality in immunocastrated pigs. J. Anim. Sci..

[41] Flores M., Armero E., Aristoy M.C., Toldra F. (1999). Sensory characteristics of cooked pork loin as affected by nucleotide content and post-mortem meat quality. Meat Sci..

[42] Van Laack R., Stevens S.G., Stalder K.J. (2001). The influence of ultimate pH and intramuscular fat content on pork tenderness and tenderization. J. Anim. Sci..

[43] Teye G.A., Sheard P.R., Whittington F.M., Nute G.R., Stewart A., Wood J.D. (2006). Influence of dietary oils and protein level on pork quality. 1. Effects on muscle fatty acid composition, carcass, meat and eating quality. Meat Sci..

[44] Ryu Y.C., Rhee M.S., Kim B.C. (2004). Estimation of correlation coefficients between histological parameters and carcass traits of pig longissimus dorsi muscle. Asian Austr. J. Anim. Sci..

[45] Rehfeldt C., Kuhn G. (2006). Consequences of birth weight for postnatal growth performance and carcass quality in pigs as related to myogenesis. J. Anim. Sci..

[46] Rehfeldt C., Fiedler I., Dietl G., Ender K. (2000). Myogenesis and postnatal skeletal muscle cell growth as influenced by selection. Livestock Prod. Sci..

[47] Renand G., Picard B., Touraille C., Berge P., Lepetit J. (2001). Relationships between muscle characteristics and meat quality traits of young Charolais bulls. Meat Sci..

[48] Karlsson A., Enfält A.C., Essén-Gustavsson B., Lundstrom K., Rydhmer L., Stern S. (1993). Muscle histochemical and biochemical properties in relation to meat quality during selection for increased lean tissue growth rate in pigs. J. Anim. Sci..

[49] Ryu Y.C., Lee M.H., Lee S.K., KIM B.-C. (2006). Effects of muscle mass and fiber type composition of longissimus dorsi muscle on postmortem metabolic rate and meat quality in pigs. J. Muscle Foods.

[50] Park S.K., Gunawan A.M., Scheffler T.L., Grant A.L., Gerrard D.E. (2009). Myosin heavy chain isoform content and energy metabolism can be uncoupled in pig skeletal muscle. J. Anim. Sci..

[51] Kim G.D., Jeong J.Y., Jung E.Y., Yang H., Lim H., Joo S. (2013). The influence of fiber size distribution of type IIB on carcass traits and meat quality in pigs. Meat Sci..

[52] Hamill R.M., McBryan J., McGee C., Mullen A.M., Sweeney T., Talbot A., Cairns M.T., Davey G.C. (2012). Functional analysis of muscle gene expression profiles associated with tenderness and intramuscular fat content in pork. Meat Sci..

[53] Listrat A., Lebret B., Louveau I., Astruc T., Bonnet M., Lefaucheur L., Picard B., Bugeon J. (2016). How muscle structure and composition influence meat and flesh quality. Sci. World J..

[54] Realini C.E., Vénien A., Gou P., Gatellier P., Perez-Juan M., Danon J., Astruc T. (2013). Characterization of Longissimus thoracis, Semitendinosus and Masseter muscles and relationships with technological quality in pigs. 1. Microscopic analysis of muscles. Meat Sci..

[55] Oury M.P., Dumont R., Jurie C., Hocquette J., Picard B. (2010). Specific fiber composition and metabolism of the rectus abdominis muscle of bovine Charolais cattle. BMC Biochem..

[56] Solomon M.B., Lynch G.P. (1988). Biochemical, histochemical and palatability characteristics of young ram lambs as affected by diet and electrical stimulation. J. Anim. Sci..

[57] Harrison A.P., Rowlerson A.M., Dauncey M.J. (1996). Selective regulation of myofiber differentiation by energy status during postnatal development. Am. J. Physiol. Regul. Integr. Comp. Physiol..

[58] Poleti M.D., Regitano LC A., Souza G.H.M.F., Cesar A.S.M., Simas R.C., Silva-Vignato B., Oliveira G.B., Andrade S.C.S., Cameron L.C., Coutinho L.L. (2018). Longissimus dorsi muscle label-free quantitative proteomic reveals biological mechanisms associated with intramuscular fat deposition. J. Proteomics.

[59] Bergeron K., Julien P., Davis T.A., Myre A., Thivierge M.C. (2007). Long-chain n-3 fatty acids enhance neonatal insulin-regulated protein metabolism in piglets by differentially altering muscle lipid composition. J. Lipid Res..

[60] Brownsey R.W., Boone A.N., Elliott J.E., Kulpa J.E., Lee W.M. (2006). Regulation of Acetyl-CoA Carboxylase.

[61] Jensen-Urstad A.P.L., Semenkovich C.F. (2012). Fatty acid synthase and liver triglyceride metabolism: Housekeeper or messenger?. Biochim. Biophys. Acta BBA Mol. Cell Biol. Lipids.

[62] Wang H., Wang J., Yang D., Liu Z., Zeng Y., Chen W. (2020). Expression of lipid metabolism genes provides new insights into intramuscular fat deposition in Laiwu pigs. Asian Austr. J. Anim. Sci..

[63] Duran-Montgé P., Theil P.K., Lauridsen C., Esteve-Garcia E. (2009). Fat metabolism is regulated by altered gene expression of lipogenic enzymes and regulatory factors in liver and adipose tissue but not in semimembranosus muscle of pigs during the fattening period. Animal.

[64] Scheeder MR L., Gläser K.R., Eichenberger B., Wenk C. (2000). Influence of different fats in pig feed on fatty acid composition of phospholipids and physical meat quality characteristics. Eur. J. Lipid Sci. Technol..

[65] Bee G., Gebert S., Messikommer R. (2002). Effect of dietary energy supply and fat source on the fatty acid pattern of adipose and lean tissues and lipogenesis in the pig. J. Anim. Sci..

[66] Fang J., Xu H.J., Liu Z.Q., Gu W., Tang B., Wang Y., Yin R., Fan M.Z. (2010). Effects of dietary inclusion of soy oil on growth performance, carcass characteristics, serum metabolites, hormones and meat quality in finishing pigs. J. Food Agric. Environ..

[67] Gao Y., Zhang Y.H., Zhang S., Li F., Wang S., Dai L., Jiang H., Xiao S., Liu D., Sun B. (2011). Association of A-FABP gene polymorphism in intron 1 with meat quality traits in Junmu No. 1 white swine. Gene.

[68] Damon M., Louveau I., Lefaucheur L., Lebret B., Vincent A., Leroy P., Sanchez M.P., Herpin P., Gondret F. (2006). Number of intramuscular adipocytes and fatty acid binding protein-4 content are significant indicators of intramuscular fat level in crossbred Large White× Duroc pigs. J. Anim. Sci..

[69] Gerbens F., Rettenberger G., Lenstra J.A., Veerkamp J.H., Te Pas M.F.W. (1997). Characterization, chromosomal localization, and genetic variation of the porcine heart fatty acid-binding protein gene. Mamm. Genome.

[70] Glatz JF C., Schaap F.G., Binas B., Bonen A., van der Vusse G.J., Luiken J.J.F.P. (2003). Cytoplasmic fatty acid-binding protein facilitates fatty acid utilization by skeletal muscle. Acta Physiol. Scand..

[71] Haunerland N.H., Spener F. (2004). Fatty acid-binding proteins–insights from genetic manipulations. Prog. Lipid Res..

[72] Joint FAO, World Health Organization (1985). Energy and Protein Requirements: Report of a Joint FAO/WHO/UNU Expert Consultation [Held in Rome from 5–17 October 1981.

[73] Wang R., Yang C., Song H. (2012). Key meat flavour compounds formation mechanism in a glutathione–xylose Maillard reaction. Food Chem..

[74] Wu G., Bazer F.W., Burghardt R.C., Johnson G.A., Kim S.W., Knabe D.A., Li P., Li X., McKnight J.R., Satterfield M.C. (2011). Proline and hydroxyproline metabolism: Implications for animal and human nutrition. Amino Acids.

